# Gender and stigma in antiretroviral treatment adherence in Mozambique: A qualitative study

**DOI:** 10.1371/journal.pgph.0003166

**Published:** 2024-07-15

**Authors:** Kirsi Viisainen, Marion Baumgart dos Santos, Ute Sunderbrink, Aleny Couto

**Affiliations:** 1 Department of Global Health and Social Medicine, King’s College, London, United Kingdom; 2 Independent Consultant, Maputo, Mozambique; 3 GFA Consulting Group, Hamburg, Germany; 4 Directorate of Public Health, Program for Sexually Transmitted Diseases and HIV, Ministry of Health, Maputo, Mozambique; London School of Hygiene & Tropical Medicine, UNITED KINGDOM OF GREAT BRITAIN AND NORTHERN IRELAND

## Abstract

Both gender and HIV stigma are known to contribute to poor retention to antiretroviral therapy (ART), but little is known how they interact in decisions about adherence or default by people living with HIV (PLWH). This qualitative study explored HIV stigma and gender interaction in PLWH’s care decisions in Mozambique. Transcribed data from semi-structured interviews of 68 men and 71 women living with HIV, were coded and analyzed for themes of HIV stigma, gender norms and treatment continuation and interruption pathways, using both deductive and inductive coding approaches. Stigma experiences were found to be influenced by gender roles. Anticipation of stigma was common across the narratives of PLWH, while women had more experiences of enacted stigma, particularly by their intimate partners. Women’s treatment interruptions were influenced by fear of partner’s negative reaction. Men’s narratives showed internalized stigma and delayed treatment due to anticipated stigma and masculine norm of strength. Severe internalized stigma was found among single mothers, who without economic or moral support defaulted treatment. Women’s pathway to adherence was facilitated by their caregiver role and support from partner or kin family. Men’s adherence was facilitated by experience of severe symptoms, provider role and by support from their mother or partner. Results indicate that linkage of stigma to gender roles interact in treatment decisions in three main ways. First, HIV stigma and unequal gender norms can work jointly as a barrier to adherence. Secondly, those resisting restrictive gender norms found it easier to manage HIV stigma for the benefit of treatment adherence. Thirdly, some gender norms also facilitated adherence and stigma management. Programs targeted at HIV stigma reduction and improving ART adherence among heterosexual populations should be built on an understanding of the local gender norms and include socially and culturally relevant gender sensitive and transformative activities.

## Introduction

Decision making over antiretroviral treatment (ART) continuation or interruption by people living with HIV (PLWH) has fundamental consequences for the success of national HIV programs and the success of the Political Declaration on HIV and AIDS adopted by UN member states in 2021 [1] by which countries aim to ensure universal access to ART to all PLWH and further to ensure that those PLWH on ART reach undetectable levels of viral load.

Despite improved access to ART, poor retention to care by PLWH continues as a concern, threatening the effectiveness of the global HIV response [2, 3]. In low and middle income countries the risk of becoming lost to follow-up from ART has been linked to three types of determinants: sociodemographic (male sex, older age, being single, unemployment, lower educational status); clinical (advanced WHO stage, low weight, poor functional status); and behavioral (poor adherence, nondisclosure) [4]. Despite the accumulated knowledge on determinants of retention to ART, there is still little knowledge on the mechanism by which stigma and gender roles, the two significant factors individually found to be linked to adherence, interact in patients’ care decisions.

Sub-Saharan Africa carries the highest burden of HIV and poor adherence to ART there has been linked to quality of health services, treatment related costs, the need to maintain social support networks, stigma, as well as to reliance on traditional medicine [5]. Studies from Zimbabwe [6, 7] and Uganda and Kenya [8–10] have indicated that decisions about ART by PLWH were also influenced by the local gender norms which did not equally empower men and women to decide about treatment initiation or continuation. Studies in Mozambique have shown a poorer negotiation situation of women in decision making over HIV prevention during pregnancy and breastfeeding period [11–14], but there is scarcity of studies on gender norms in PLWH decisions about adherence and default.

HIV stigma has been shown to contribute to poor ART adherence across various socio-cultural domains at personal, relationship and structural levels, primarily through undermining social support and adaptive coping [15], with increased mental health vulnerability and reduced self-efficacy as suggested mechanisms [16]. HIV stigma experience has manifested a gender difference in various studies in sub-Saharan contexts. Measured with the HIV Stigma Index women experienced higher levels of stigma than men in Kenya [17] and a small qualitative study suggested HIV stigma to have a more extensive negative impact on women than men in Ghana [18]. HIV status disclosure and stigma have been linked to gender specific effects on HIV risk taking behavior [19]. Voluntary HIV disclosure has shown also substantial gender differences in HIV care engagement in a context of universal ART availability in Kenya and Uganda, where unmarried men more often were not disclosing, not using condoms nor engaging in treatment; while married men were found to disclose to their partner more often than married women who feared abandonment or violence, and were more often disrupting their treatment [20]. In Mozambique HIV stigma has been linked to men’s lower uptake of testing [21] and generally to disclosure and treatment initiation [22], but studies on HIV stigma and ART adherence are lacking.

This paper adds to understanding of the mechanisms by which HIV stigma experience and gender roles interact in ART care decisions and adherence in Mozambique. Findings can be used to document the challenges faced by PLWH on treatment and to develop interventions for improving adherence and retention in ART care.

## Key concepts and framework

### HIV stigma

Social studies on stigma in health build on Goffman’s seminal conceptualization of stigma as an outcome of social interaction where a negative social meaning is attached to an individual or a group that is perceived to have a “spoiled social identity” or a “discredited status” in comparison to a social norm. The source of stigmatization of individuals or groups can be physical as in case of a visibly debilitating illnesses, moral as in case of unacceptable behavior or social as in case of belonging to a certain occupation or ethnicity [23]. HIV stigmatization has developed from physical isolation of those affected due to fear of contamination in the early years of epidemic to marginalization of PLWH on more ambiguous moral and social grounds [24]. While social interactions between individuals reproduce stigmatization, HIV stigma is also tied to social conditions that structurally exclude individuals and groups from equal participating in social life, emphasizing the imbalance of power and discrimination of already marginalized groups [25, 26]. The socio-cognitive orientation of stigma studies provides three useful categories for classifying the HIV stigma experience of PLWH as enacted (experience of prejudice or discrimination), anticipated (expectation or fear of enacted stigma) and internalized (endorsed negative feelings about people living with HIV and applied on self) [27]. In this study the stigma experiences of PLWH were classified following the socio-cognitive framework as explained in detail below under themes and analysis.

### Gender

Gender in social studies refers to culturally and socially prescribed influences on behavior, role, rights and responsibilities of individuals based on their maleness or femaleness [28]. In this study we followed the definition of gender roles as social roles, encompassing a range of behaviors and attitudes that are socially prescribed to be acceptable, appropriate, or desirable for males and females in the study context. These are generally centered on conceptions of masculinity and femininity and shaped by the perceptions of what it means to be a “real man” and a “real woman” which in turn have been modified by cultural, political, class and economic influences over time [29]. Gender roles are tightly linked to behavioral norms that societies, or groups within societies, apply on males and females influencing and shaping or even controlling their everyday actions, expectations and experiences, manifested in a range of ways from dress and mannerisms to opportunities in education and labor force over life course, resulting in gendered health behaviors, challenges and outcomes [30]. Gender norms and roles are context specific, but not constant, evolving over time [31]. Gender relations refer to interactions between and within masculinities and femininities at interpersonal and structural levels [32]. In this study the interviewees’ gender references are examined as reflections of local gender norms and roles.

### Conceptual framework

**Fig 1:**
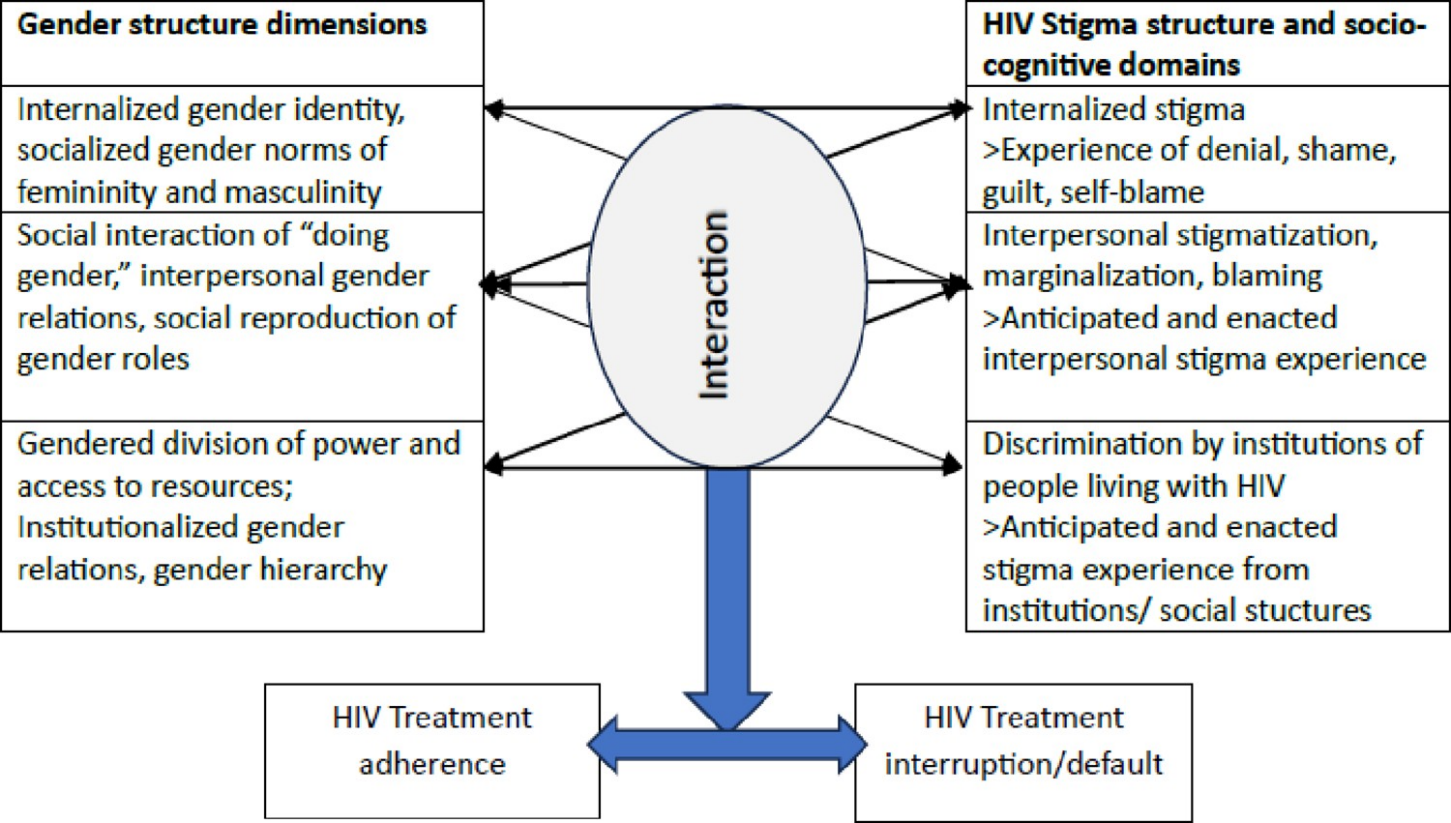
Conceptual framework for gender and HIV stigma interaction. Framework adapted from Wyrod (2013) [33].

To conceptualize the interaction between HIV stigma and gender we adapted the framework developed by Wyrod (2013) that presents the intertwined social processes of gender and AIDS stigma [33] (Fig 1). The categorization of gender dimensions follows a framework of gender system adapted from Heise et al. (2019) where gender is conceptualized at three levels as a socialized gender identity, as an interpersonal relation and situated within an established power hierarchy [30]. The categorization of HIV stigma domains follows the socio-cognitive perception classification by Earnshaw [27, 34] combined with Parker and Aggleton’s view of structural power hierarchies in discrimination [26]. The research examines the interaction mechanisms of these structural systems at the level of gender norms and perceived stigma in individual narratives about HIV treatment adherence and default.

## Study context

### ART retention and adherence in Mozambique

Mozambique has a generalized HIV epidemic, 12.5% of the adult population or 2.4 million people were estimated living with HIV in 2022 [35]. Women are affected more than men, HIV prevalence was two to three times as high among females as males in the age group of 15–19 years in 2021 prevalence survey [36]. Gender disparity has been prominent in the epidemic profile since the start of national HIV prevalence data collection, and previous studies have considered the following factors to contribute to it: poverty, poor level of education, inadequate health system coverage and unequal gender norms [37].

Poor retention in ART has been a concern throughout the scale-up of the HIV treatment program since 2004. National cohort-based data showed ART retention rates among adults at 12 months after treatment initiation to remain constantly below 70% in 2015–2019, reaching 67% in 2019, with young women and men under 30 having lowest retention rates [38, 39]. Studies on ART adherence in Mozambique have indicated lack of economic means of patients, poor quality of care at the facility, poor treatment literacy, enacted and internalized stigma of HIV, and preference of traditional medicine as barriers to treatment adherence [40, 41]. HIV infection has been shown to be a heavily stigmatized condition in Mozambique [22, 37, 42] with an indication of a gendered stigma effect on HIV testing [21].

Interventions aiming to improve the poor retention rates were developed from the National STI and HIV Program’s detailed analysis of patient flows and care practices in the facilities. They targeted to improve quality of care and access to services through differentiated care options. These included multi-month, family group, and community adherence support group dispensing of ARVs, and psycho-social counselling support to all newly diagnosed, and continuing patients on ART. There is also a specific program of support services for survivors of gender-based violence. In addition, specific cadres of community health workers to support those at highest risk of defaulting were deployed by partner NGOs. These cadres included mentor mothers addressing specific support needs of pregnant and breastfeeding women [43, 44] and lay extension workers called *activistas* making calls and home visits to men and non-pregnant women missing their appointments [45]. In 2020 the *activistas* and mentor mothers managed to bring back to care 84% of patients assigned to them [46].

### Gender roles in Mozambique

Mozambican gender roles have been marked with a strong tradition of patriarchy both in the more rural northern part [47, 48] as in the faster urbanizing southern part of the country [49, 50]. Within nuclear and extended families, women are responsible for most chores related to caring for and raising children, while men are expected to be decision-makers in households, including health matters such as use of contraception and covering costs of health care [48]. The relation between motherhood and social status is still very present in all parts of Mozambique; pregnancy is perceived as a rite of passage to adulthood by young women and motherhood as a means of achieving social status by adult women [11, 12]. Limited economic and educational opportunities for young women as well as the desire to be considered a “grown-up woman” (*mãe*) increase pressure to early sexual relations, pregnancies and marriages: 36 per cent of adolescents aged 15–19 had been pregnant at least once in the 2023 national demographic and health survey [51].

The traditional male household head role has been based on a combination of men’s ability to uphold their status and role by their control of agricultural production and income, and by responsibility and authority over women and children [52]. The socially recognized ideal of manhood in Mozambique is to have multiple lovers or wives, and to provide for the family, as well as their lovers [47, 53]. In situations of extreme poverty, economic hardship hinders living up to the provider ideal causing stress of loss of respect and authority at home [53]. Even with changes in economic activities, being able to provide for the family still is one of, if not the main, determinant of the male gender role throughout the country. The traditional notion of responsibility for and power over women and children is intimately connected with a view of male sexuality that allows men a high degree of sexual freedom especially in urban settings, and in rural polygamous communities. Expectation of men living up to an ideal of virile masculinity increases their vulnerability to HIV through risky behavior [52]. In a gender equality survey women in Maputo defined a “good woman” by her ability to have patience with her husband having another sexual partner [53]. Answers to the latest demographic and health survey where four and 36 per cent adult women and men in Mozambique respectively reported two or more sexual partners in the last 12 months [51] also reflected the socially acceptable gendered behavior.

## Subjects and methods

### Study sites and subject selection

This paper presents a targeted analysis of individual HIV patient interviews conducted for a qualitative study aimed to contribute to a better understanding of patient-related reasons underlying the low ART retention rates among adult patients on ART in Mozambique (parent study). The site selection for the parent study was purposefully seeking areas with the lowest reported retention rates. Twenty-nine districts provided 75% of the reported lost to follow-up cases in 2018. For the parent study 21 of these districts were clustered into three sub-regions: the central Zambezia Province, the northern Nampula Province and the southern Maputo City and Province. Two districts with the lowest reported retention rates in 2018 (ranging between 56% and 67%) in each sub-region were selected, and in each district the health facility with the highest number of patients on ART (ranging between 4,115 and 19,194 patients) was selected for the research team to interview patients and staff. Further, one district with a high retention rate (82%) was also included in the central sub-region.

The parent study applied a qualitative design, using semi-structured interviews with people living with HIV, and focus groups with health workers. The selection of the interviewees was a mix of purposeful and convenience sampling. Patients who were known to have defaulted by their medical records were sought to be interviewed, purposefully including adult men and women under 30 years of age, known to have the highest risk of defaulting. The Mozambican public health system definitions of treatment default were used: a patient was considered as lost to follow-up by not collecting their ARVs in 60 days or more since their last appointment for pharmacy refill and are still alive and not in treatment elsewhere. 72 patients with a known history of defaulting treatment were interviewed at their homes accompanied by the local *activista*. To identify differences between defaulters and those who were compliant, a convenience sample of 80 people reported as active on treatment in the same facilities was also interviewed. They were selected based on the medical records of the list of patients on ART on the days of the research team’s visit to each study site.

The individual interview data from the parent study was considered suitable to merit additional more detailed analysis for the research question of this study as the interview questions included stigma, gender roles and recall of adherence in care and reasons for any interruptions. The selection of sites included northern, central and southern districts covering the regional differences in gender roles.

### Data collection and analysis

Interview data was collected between 19 October 2020 and 9 December 2020. Semi-structured interview guides were used in the individual interviews (S1 File). The interviews, that lasted between 20 and 45 minutes, focused on the contextual factors such as access and perceived quality of health care, experience of living with HIV, gender norms and perceived stigma, as well as on individual factors such as access to family support, self-motivation capability, self-esteem, and individual resilience. All interviews were conducted in Portuguese by two local resident researchers (one of them MBS), recorded and transcribed verbatim. On-site interpretation to and from local languages was conducted when requested by interviewees. In these cases, the transcribed files included the answers interpreted in Portuguese, not the original spoken local language.

For the analysis of this paper the interview transcripts of individual people living with HIV were manually coded for the themes of stigma and gender roles using both deductive and inductive coding approaches. Coding was done by two qualitatively trained researchers: MBS coded the data for the parent study report using Nvivo software, and KV coded the interviews for stigma, gender and adherence and default references manually for this paper. MBS reviewed the coding by KV against the parent study codes and any differences were discussed and agreed. No analysis software was used in the coding process for this paper, coded quotes were marked in transcript files and grouped by codes in excel files for analysis.

### Concepts and themes in analysis

In the analysis we followed the socio-cognitive framework for the categorizing of the stigma experiences of the interviewed PLWH as enacted (experience of prejudice or discrimination) with sub-codes of own experience and witnessing experience of prejudice by others, anticipated (expectation or fear of enacted stigma from others), with sub-codes of expectation from family, from close neighbors or workmates, or from community in general, and internalized (endorsed negative feelings about people living with HIV and applied on self) [27]. Following the UNAIDS stigma index tool definition of internalized stigma we included interviewees’ mentions of denial, shame, guilt and low self-esteem as sub-codes of expressions of internalized stigma [54]. These were used as *a priori* codes in coding stigma experiences. but no scales of stigma severity were used.

Further, we defined gender roles as social roles, encompassing a range of behaviors and attitudes that are socially prescribed to be acceptable, appropriate, or desirable for males and females in the study context [29]. For Mozambique we used thematic categories of traditional gender roles arising from previous studies for *a priori* codes of female gender roles: women’s normative role as carer, provider of offspring, dependent on husband’s financial support, obedient to husband’s decisions and maintaining her attractiveness [47, 49, 50, 52, 53, 55]. Men’s traditional normative role according to the literature was coded to have strength to work and provide for family, have authority in decision making in family and to show sexual vigor by having lovers [52, 53, 55].

Treatment adherence was defined purely by interviewee’s recall of compliance to clinic appointments and of taking the prescribed medication. Adherence was classified to categories of active (no history of continuous treatment interruptions of more than 60 days) and defaulted (history of interruptions lasting 60 days or more) based on the interviewee’s recall of their medication compliance.

Transcripts of both defaulted and active interviewees were first coded for occurrence of references to gender roles and categories of stigma experience. Further we sought for emerging patterns how these interacted in the treatment narratives either as supporting or hindering treatment interruptions.

### Ethics statement

The study received ethical approval from the National Bioethics Committee for Health within the Ministry of Health in Mozambique on April 23, 2020. There were no deviations from the study protocol after obtaining approval. The purpose of the study was explained to all the identified patients, and their informed consent to be interviewed was attained in writing from those literate in Portuguese, and through a recorded verbal consent from others. One of 152 identified and interviewed patients refused recording, and 12 interviews were discarded from this analysis due to an incomplete recording and transcript.

## Results

### Characteristics of the study participants

The study sample for the re-analysis included 139 successfully recorded and transcribed individual interviews. There were 71 women and 68 men among the study interviewees, the majority of whom were either married or in civil or traditional partnership (Table 1). There were 31 single women within the study group, 19 of them were also single heads of household. Forty-four of the interviewees did not know their own age. Based on interviewees’ own description of their adherence the interviewees were re-classified as 77 continuously active on treatment and 62 with history of default of over 60 days. Among the defaulters there were three women and six men who at the time of interview had been without ART medication for over 60 days (ranging from five months to two years) and not returned to care. All other defaulters had a history of one or more treatment interruptions of a minimum of 60 days but had since returned to care. 17 of the 77 interviewees classified as active (9 women and 8 men) also self-reported history of treatment interruptions shorter than 60 days.

**Table 1:** Characteristics of study sample.

	Women (n = 71)		Men (n = 68)		Total (N = 139)	
**Adherence**						
Active*	40	(54%)	37	(46%)	77	(100%)
Defaulted > 60 days	31	(56%)	31	(44%)	62	(100%)
Age**						
16–29	28	(67%)	14	(33%)	42	(100%)
30–70	29	(49%)	30	(51%)	59	(100%)
Does not know	20	(57%)	24	(47%)	44	(100%)
**Civil status **						
Single or separated	31	(61%)	20	(39%)	51	(100%)
Married or cohabiting	40	(45%)	48	(55%)	88	(100%)
**Region **						
Northern	18	(46%)	21	(54%)	39	(100%)
Central	30	(52%)	28	(43%)	58	(100%)
Southern	23	(55%)	19	(45%)	42	(100%)

*Reporting no interruptions, or less than 60 days of treatment interruption duration.

**Self-reported age in home interviews, in clinic interviews verified from records.

Religion of interviewees was not specifically asked, but according to 2019 government census data majority of population in the northern region was Muslim, while in the southern and central regions people were predominantly Christian [56]. The two southern study sites and one northern site were health facilities in city suburbs with a mixed population of traders, casual laborers, and unskilled workers. One central site was fully rural where people are subsistence farmers and spend part of the year in the fields outside of their village, sometimes at a considerable distance. The three central and northern sites were partly urban partly rural districts. The study sample reflected these general socioeconomic and religious characteristics of the regions with most of the participants being self-employed either subsistence farmers, traders, or casual day-laborers or unemployed. There were only a few participants in salaried occupations such as teachers, drivers, or agricultural technicians in the sample.

### Gender references in the PLWH interviews

#### Female gender role

Female gender role categories and examples from the interviews are collected in Table 2. Women’s narratives included several references of early first partnership and childbearing in teen years, particularly in the northern region. Women considered important to have a partner and to bear children from each partner. Women did not consider the HIV was affecting their core femininity, the ability to have children, some even made the point that being on treatment will make them look healthier, fatter, and more fertile. Women’s narratives commonly included several subsequent partners with pregnancies from each partner and a wish for a new partner if separated. A few women in the north reported participating in a polygamous household, some others referred to their partners having other wives or lovers in another household. In their obedient role most women referred to their husbands’ taking decisions about treatment and about separation, as a matter of fact, without contesting them. In addition to the *a priori* codes from previous literature the role of older women as respected advisers emerged in interviews of both men and women who consulted and obeyed their mothers in treatment decisions, and in marital disagreements. A few women resisted the gender norm and showed independence from husband’s restrictive decisions by leaving them, or by continuing treatment against husband’s will, usually with support from their birth family (Table 2).

**Table 2:** Interview examples of female gender role.

Main code: Femininity, normative female role	Examples from women’s interviews*.
Subcode: Healthy women are feminine, attractive, fat	You see that so-and-so is fat, she’s pretty because she’s on medication, if you behave badly, you don’t medicate or like you don’t eat well, you’ll have a bad body. (Kamavota E)
	But I can see they’re even fat, really fat, until I tell you that this one has HIV/AIDS, you don’t believe it. (Morrumbala 2)
	(Interpreter:) She feels like a woman. With the medications she is taking she is even more robust. (Nampula D)
Subcode: Bearing children is important for womanhood	It is every woman’s dream to be a mother, when the time comes, I’ll have one. (Kamavota 7).
	I didn’t finish the tenth grade. It was during that time that I became pregnant…with my daughter. I want to continue treatment to be able to take care of my children. (Nampula 9)
	(Interpreter:) She continues taking it [medicine] because she has noticed that then the child is HIV-negative, this is good for her, she can continue to give birth. She feels like a woman. With the medications she is taking, she is more robust. (Nampula D)
Subcode: Husband important as provider and for status	After the death of my [first] husband, and as a woman I have depended on men, I had the opportunity to marry another man to support myself and have two more children with him. (Morrumbala 9)
	I think it is necessary to have a partner just for me, because as he has to support two households it is almost not enough to help, you see, so if I had a partner just to look after my house, everything would be solved. (Nicoadala 3)
Subcode: Obedience to husband’s decisions	I don’t know if my husband has it [HIV] or not. He didn’t give any reason [for not testing], so much so that I didn’t force him to [tell]. (Nicoadala 12)
	I talked to him [about condoms use], he said he can’t stand it, can’t do it, yeah, told me to leave it, nothing there. From there on, that is the way we stayed. (Nicoadala 3)
	I didn’t oblige him [to go to test] as much, as he also has his first wife. I think he already did [the test] with the first one. (Namacurra E)
	But he found out [about my treatment] on his own, he asked what [the pills] were and I said ‘I’m on medication, now I don’t know, what do you decide?’ (Nacala D)
Subcode: Women are carers	In addition to my own children, I have a child who has no father or mother. (Morrumbala 9)
	Born today, tomorrow the mother dies and the child suffers. So, when I started to think about that and think about my children, I said ‘no, I have to go back to treatment, because I have to see them grow up‘, yes. (Morrumbala 2)
	I count on my wife’s help, there are times when I’m not in a good way and she also comes to collect [medicine] for me. (male, Nacala 17)
Subcode: Respect adviser role of mother, mother-in-law and older members of family	I spoke to my grandmother, because she is a peer educator; my mother’s mother. She encouraged me to undergo treatment. (Kamavota N)
	If I neglect my life, my mother will have one less [child] and my mother is the only mother I have. (male, Kamavota I)
	My motivation for not giving up on medication is the advice I have received from my family. They say that ’you are single, you don’t have a husband. If you get sick due to giving up on medication, you won’t have the strength to go to the fields to produce and feed your children.’ (Namacurra 2)
	At that time I could call my older sister, to inform her of the [HIV diagnosis], and she advised me that ‘my sister, for you to maintain a good and healthy life, you have to listen to the advice [of the hospital] and take [medicine] according to their recommendation’. (Morrumbala 9)
	[The] only person is my mother who knows, yes. She demanded that I don’t give up, right? Yes, she demanded that ‘Look, you can’t give up. It is like that. You must take [the medicine]; you must follow all the rules they tell you.’ (male, Nampula 6)
**Main code: Resisting normative female role**	
Subcode: Women resisting men’s authority	My first husband forbade me to take medicine, but I did not listen to him. I was told at the health service not to listen to him, this is my health. (Morrumbala 10).
Subcode: Women resisting importance of married status	I don’t know if he knows too and doesn’t want to tell either, but I’m going to start, to tell him, to say, hey, that’s it, if you want to stay with me, stay, because the men are like, when they know something like that oh no, it’s not me,. . . .heh no, so hey, I’ll face it, I’d rather lose [him] but at least I already told him. (Kamavota 9).

*Quotes from men indicated specifically.

#### Male gender role

Men’s gender role coding categories and examples from interviews are in Table 3. Men’s narratives presented a decisive role in defining themselves whether they were sick or not, particularly when tested without symptoms. Many of them initially doubted or denied test results and required a lot of convincing by health staff or older birth family members to accept their diagnosis and start treatment. If they experienced grave illness symptoms, they were more easily convinced of the diagnosis. Men did not want to discuss illness or share about it beyond people they could trust keeping the secret, such as their own mother, and usually also their wife. Men also emphasized their provider role which was either an incentive to stay healthy and strong and on treatment, or a constraint to adherence when work travel or working the field was in conflict with clinic visits. There were a few men who mentioned being polygamous, and some of the married men referred to their current concomitant lovers, or to having had several relationships earlier in line with the masculine ideal of virility. A few men resisted the normative male role by reducing the number of lovers after diagnosis or by opening up to their friends about their HIV diagnosis.

**Table 3:** Interview examples of male gender role.

Main code: Masculinity, normative male role	Example quotes from men’s interviews*.
Subcode: Men are strong.	I miss the body *epah* I really do, I wasn’t like this. I don’t know what it is but, hey, I really lost my body. I feel bad. A young man like me to lose strength, *epah*. (Nampula G)
	That’s what I started with, I started to classify my body weak because I’m not like I used to be. So that’s what almost makes me think about going back there for treatment. (Nampula G)
	Now I really am a man–a strong man! Yes! (NIcoadala B)
Subcode: Men don’t discuss illness.	What I think, it is a disease, you don’t have to keep saying that I have a disease. (Nacala 17)
	We men are not women, it’s very difficult for us men to sit down and open up, talk about it [disease]. When we wake up, we only take our machetes with us to do our jobs. (Namacurra 7)
	There is no need for me to go to a friend and talk for a while about my life or talk about my issues. I can’t do it there. (Nampula L)
	At work there is no discussion [about HIV} either, but if anyone has it maybe they tend to hide it like I do. I also hide it because I can’t say I have it. (Morrumbala J)
	When someone is [HIV] positive, there in the neighborhood, they don’t talk and just shut up, each one knows for himself. (Morrumbala 12)
Subcode: Men don’t admit being ill.	[The men] do not want to tell the truth [about their HIV status], do they? (female, Namacurra 13).
	What took me to the hospital was not something to do with AIDS, it was simply malaria. How come I go for one illness, and they say I have another? So, I didn’t receive it in a good way. (Namacurra H)
	I saw many people die, who were my companions, colleagues because of their own sloppiness. (Kamavota I)
Subcode: Men’s authority in family decisions.	I always oblige her[wife]–when I come for a checkup, she also has to go for a test and give me the result (Nampula 4).
	I have to stay, not to mess up, meet him [husband] to see how he will react later [to the HIV diagnosis]. (female, Nicoadala 3)
Subcode: Men provide for the family.	I started to see that the days I was coming to the hospital I had to be losing my daily bread. (Nacala 17)
	Time is worse for us, sometimes a whole day, for us who are traders, losing a day for us is a big loss. (Nampula G)
	I’m not sick, but I have a virus, it doesn’t stop me from doing my work (Namacurra 7).
	My financial responsibility at home was suffering because I was sick. But when I started taking medication my strength recovered and now, I am already supporting my home. (Morrumbala 8)
Subcode: Men have many partners or lovers.	It was my free will to test because I had many girlfriends, many mistresses. So, I suspected myself because I had used any woman. I really wanted to know the state of my body but the result scared me. (Namacurra 10)
	For me, the issue of masculinity, given [my] age, it’s not as relevant as when I was young, when I was at a very sexually active age, which I really liked. For me it’s an issue of sports, if someone comes along who likes it, then I’m sexually involved but it’s not because it’s an issue that really affects my manhood as such. (Nicoadala 4)
	My head was a little sick, it hurt a lot. . . and I immediately suspected that it was HIV. . . I had been with women a bit … my wife was already taking [ART} at the time. (Kamavota 1)
	My late husband was young. Despite being young, he had already ’polygamized.’ He already had other women besides me. (female, Namacurra 2)
**Main code: Resisting normative male role**	
Subcode: Stopping having many lovers.	Much more, I just reduced the way I played, I stopped a little, I don’t have those games that I had there before, days ago, I changed. (Kamavota 1)
	I never had sex with any other woman [but wife] again [after diagnosis]. (Nacala 13)
Subcode: Speaking openly about own illness in community.	Disease is disease. There is no point in hiding that the person is sick. I speak willingly, no problem because I’m not afraid, I know I’m healthy through this [treatment]. (Chamanculo 5)
	I speak {about my HIV] with a lot of people, for example, my neighbor friends. (Kamavota A)

*Quotes from women indicated specifically.

### Stigma in PLWH interviews, both female and male

Anticipated stigma was the most expressed form of stigma in the interviews. Both women and men feared being labelled with HIV/AIDS, isolated, rejected, laughed at, or gossiped about in the community. The interviewees managed exposure by keeping the diagnosis and treatment a carefully guarded secret among trusted family members and selected friends and in a few cases just for themselves.

Interpreter: She prefers not to inform the neighbors because they start gossiping about whatever. They say that lady there has AIDS, you can’t marry her, you can’t go out with her, you can’t talk to her, she has AIDS, that’s what they’re talking about. (female, Nacala E)

Usually when I go to the pharmacy, I carry a purse, when I get there, I put the medicine in my purse and leave. Yes, that’s why I haven’t exposed myself, to avoid certain discriminations. (female, Kamavota E)

Interpreter: She said if her neighbors knew that she had HIV, they would be whispering along the way, you see,’ that one has HIV’, but now as they don’t know, she is fine. (female, Morrumbala D)

The reason I don’t want to tell my friends [about HIV] is that no secret is kept. It is enough for me to tell one friend; he will tell another friend of his; from there it will spread… insult time begins. They’re insulting me ’that’s the one…’ and then you cannot be walking free. (male, Nampula 4)

When someone is [HIV] positive, there in the neighborhood, they don’t talk, they just shut up, each one knows for himself. It’s shame, they feel shame. In order not to be laughed at by others, not spoken badly. (male, Morrumbala 12)

Seven of the women and five of the men considered that there was no bad talk of HIV positive people in their communities, but only three active women and two men were open about their diagnosis in the community, one woman was a young activist, other two were supported by a church group, and a savings group for HIV positive people. One of the men had had HIV for 16 years and learned after several interruptions to accept his diagnosis and be open with his neighbors and friends, even urging others to test.

I speak with a lot of people, for example, my neighbor friends, I’ve always been open with them, so far there are a couple who are uncomfortable, but I’m insisting with them, so that they can at least go once to do a test (male, Kamavota A).

Despite the widely feared and acknowledged existence of stigmatization direct experiences of discrimination due to HIV were rarely admitted. Only one woman spoke of her neighbors directly scolding her due to her status. Both women and men generally accounted discrimination as something that they had witnessed or heard of happening to others, not to themselves.

The neighbors discriminated, they called my auntie names on the street, or in the market. There came a time when she couldn’t even walk, I had to take her to the hospital. She leaned on me to not fall, she was too thin, beaten down. And people always saw us with a bad look. And sometimes when I passed by, they asked “are you the niece of that so-and-so who is sick, with so-and-so disease.” (female, Kamavota E)

They say that hey, the owner of the house over there isn’t even worth it to say hello to. Even the number of friends has reduced for him. The words you’ll be hearing are words that you won’t like, and can come close to losing friendship, yes. It’s really bad. (male, Nampula G)

Yet, in their narratives of diagnosis and starting treatment several women accounted that their partner had rejected or abandoned them at time of learning about their HIV diagnosis, a form of enacted stigma, although not recognized by women as such. Both knowledge and experience of abandonment by partner were common among the women, and a cause of much anticipated stigma for future relations with men.

[And how did your husband react (to your disclosure)?] Whoa! Didn’t react at all. I just told him: ‘you need to start medicating too’. He didn’t say anything, and he didn’t go to the hospital either. I just saw… him abandoning me. He just left (female, Nicoadala C).

I had a husband, when he found out [about my HIV], he abandoned me (female, Nacala 3).

I hid the medicine; I didn’t want to tell, or he would leave me. I hid it from him, but he found out. And when he found out, it didn’t cause trouble (female, Nacala D).

My partner said that he accepted my diagnosis, but I don’t know whether that is true deep down in his heart. He is negative and has stayed with me. I don’t want to shock him, and talk about it, because he might change his mind, I always tend to take the medicine when he’s not there (female, Nampula 1).

In contrast, while disclosure to intimate partner was difficult for men as well, due to internalized guilt and shame, there were very few accounts of men being abandoned by their wives due to HIV. Only one man told of his previous wife having left after his HIV diagnosis, and another explained that his HIV positive wife divorced him due to his inability to provide due to his illness. Other men found their wives to stay and support them in managing the treatment.

I was afraid to inform my wife of my diagnosis. I received my treatment card, and I hid it inside the house. But she does the cleaning and saw it, read it, and asked… I didn’t want to answer, I pretended not to know. But then she started to get sick and went to get tested and was positive. She asked me if I would agree to continue with her, and I said, “I was first”. There was a discussion… I apologized, and we both continue treatment together, thank God, we are both fat and fine. (male, Nacala F)

I was afraid because I thought I was guilty. But she was sincere because she didn’t blame me. Any other woman would be angry with me. Finally, I’m the one who brought the virus home. Yes, but not by wanting to. (male, Nicoadala 6)

Internalized stigma of HIV was expressed in different gradients both by men and women, as initial doubt and denial, which could delay the start of treatment or lead to disruption of treatment when the symptoms disappeared. In a few cases it led to complete abandonment of treatment when diagnosis did not fit in patient’s own understanding of what their problem was. Doubt and denial of the clinic’s diagnosis was more common among men.

Then they gave me the medication on the same day I took it, but I didn’t medicate, with that fear that “no, I don’t have it” because I was sure that I didn’t have it. I thought that this disease was only found among older, more experienced people (female, Kamavota E).

I think the people who usually get this disease are people who prostitute themselves, and I do not. I only had stomachache, not symptoms of HIV. In fact, I have seen people who have it because they are a little weak, I think that the person takes medication when they see that they are actually getting worse, or they are sick. Now in my case I ended up not adhering to it because I feel strong, I’m fine (female, Namacurra C).

They said you have AIDS, the HIV virus, but we argued a lot because what took me to the hospital wasn’t something connected to AIDS, it was simply malaria. When I got home I reflected, I left the HIV medication aside, I took the malaria medication, I got better and that was it (male, Namacurra H).

In this community we think that having the HIV disease is resulting from opportunism by sorcerers… from envy of neighbors (male, Namacurra K).

The internalized shame of HIV and the anticipation of stigmatization by others were often present in same interviewees, as expressions of same phenomenon, leading to strategies of non-disclosure of the state and hiding of medications, and in some cases treatment disruption.

To this day, not even my friends know that I take [ART]. [Interviewer: What are the reasons not to tell?] I think it’s shame (female, Nampula 13).

The reason for me going out with a friend and forgetting to take [the medicine], traveling to Nampula and leaving medicine here, was because I was always ashamed to carry that medicine. Right there I didn’t take it, but later I really regretted it (female, Nacala D).

When someone is [HIV] positive, there in the neighborhood, they don’t talk and just keep quiet, each one keeps it to himself. It is shame, one feels shame, not to be laughed at by others, not spoken badly about (male, Morrumbala 12).

At work there is no discussion [about HIV} either, but if anyone has it maybe they tend to hide it like I do. I also hide it because I can’t say I have it (male, Morrumbala J).

With improved awareness and access to treatment, visible signs of HIV through opportunistic infections were becoming rarer in the community, but HIV stigma was resilient to this change in the perception of PLWH. Thus, being on ART had for them become the signifier of the stigmatized state, as signs of illness had been substituted by treatment cards and medicine bottles to define who lives with HIV.

I feel bad, because the intention of other people, as I said, when they see you taking pills, their intention when they meet someone else is to point out ‘that one takes pills’. You see, while I know that I’m seen like that I can’t be happy. (female, Kamavota M)

In most severe form of internalized stigma some women and men expressed despair of their life situation, low self- esteem, even signs of depression and suicidal thoughts. All of these interviewees also had a history of prolonged periods of treatment default.

It’s one of the things that demoralizes me a lot and I, not because I’m ignoring the treatment, I want to follow so badly, I want so much to be that person like the others that medicine trusts, but I’m not able to, because of my condition, I’m not anything (female, Chamankulo D).

(Interpreter): She said that there was no reason to live, "since I’m infected, I don’t have anyone, my family doesn’t give me that attention, so it’s better for me to commit suicide" (female, Kamavota J).

Overall, the women and men spoke very similarly about their anticipated fear of HIV stigmatization by their communities. The gender differences in the stigma experience were found in the prolonged internalization of shame, guilt or denial of the HIV diagnosis by men, and women’s experience of enacted stigma in their intimate partner relationships.

To further examine how stigma was managed by women and men who remained adherent and those who defaulted treatment we collected emerging examples of pathways leading to adherence or default from the interviews.

### Pathways to treatment interruption or default

Strong denial of HIV and alternative explanations to own situation were linked to complete refusal or abandonment of treatment proposed by the health service for months or years. Examples of this strongly felt internalized stigma were in narratives of both men and women. Once they had started treatment, women’s later interruptions were influenced by a combined effect of their submissive role in partner relations and anticipated or enacted stigmatization by authoritative partner as well as economic dependence on male provider role. Treatment disruptions by women who ended in the role of a single head of household were influenced by the hierarchical position of women with less economic opportunities to support themselves and provide for their children.

**Fig 2:**
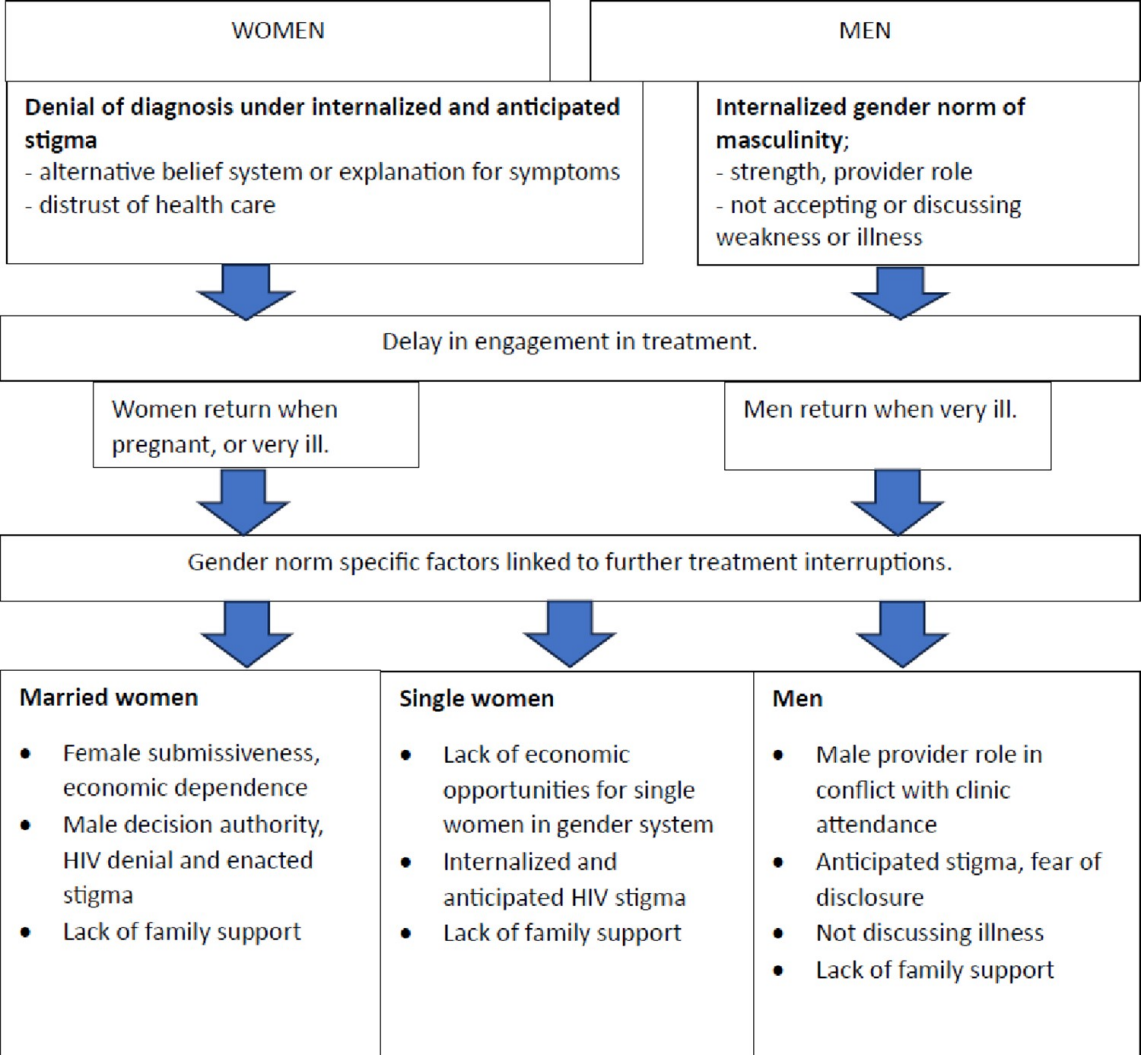
Gendered pathways to treatment interruption under HIV stigma.

In contrast, men’s pathways to treatment interruption were more often about persistent denial of the diagnosis, delay of treatment and non-disclosure of diagnosis influenced by anticipated or internalized stigma combined with the male gender norm of strength and not admitting illness. Men who had accepted and started treatment ended up having treatment interruptions when their role as family provider conflicted with clinic attendance. A summary of main gendered pathways to treatment interruption and default are presented in Fig 2, with examples in the following section.

### Women’s pathways to treatment interruption

#### Partner not accepting HIV diagnosis

In the women’s histories of managing their HIV diagnosis and treatment the pivotal issue that led to either interruptions or adherence in treatment was their partner relationship. Among married or cohabiting couples, the women were often the first ones to be tested, in HIV screening of prenatal care, family planning or other services usually without any symptoms. Many of these women delayed disclosure of HIV diagnosis or tried to hide their medicine for fear of abandonment by partner if HIV diagnosis was revealed. The importance of stable partnership for economic and status reasons fueled fear of divorce, and some women stopped treatment in order not to disclose. Without husband’s or his family’s acceptance, women found it difficult to maintain both the treatment and the conjugal partnership. A young woman in her 20s talked about her polygamous marriage, HIV diagnosis and treatment interruption:

I met my husband when I was still very young, he is a businessman here. He was my first and only man I ever knew, and he gave me a lot of gifts. I was his fourth wife, all of us lived together in the same household. When I got pregnant, I tested positive. I asked my husband to come with me to the clinic, but he refused. He didn’t want to know about HIV. So, I took the medication secretly until the baby was born. My husband prohibited me from taking the medication, he didn’t want to have to deal with it. So, after the baby was born, I stopped for several months. I wanted to preserve the marriage and was hoping that maybe the test result was wrong (Morrumbala M).

Later, however, with the support of her mother she resumed treatment and left her husband to return to her birth family.

#### Single head of household, poverty and depression

Several women counted experiences of having been abandoned by their husbands when they revealed their HIV status ending up caring for their children alone. Single motherhood meant greater vulnerability to treatment interruptions due to the uncertainty of livelihood, even to extreme poverty, and lack of supporting network. Women widowed by HIV were in particularly high risk of falling into a worsening spiral of inability to maintain livelihood and health of themselves or their children in traumatic life conditions of poverty, or to find a new stable partnership. A 30-year-old single mother of six from the central region had been diagnosed with HIV after her first husband died. Later, her second husband left her after learning of her diagnosis. She interrupted treatment for more than a year due to difficulty keeping appointments when caring for her children alone. She explained about her fears of not finding another partner and internalized stigma through an interpreter:

She said that ever since her husband left the house…no one has yet appeared to propose. She feels ashamed–she feels bad, yes. She thinks about it like *’epah*! If another man comes, what will it be like?’ She is afraid, will he want me? (Nicoadala C)

In the worst scenario, single mothers living in poverty showed signs of low self-esteem, apathy, and depression, and with no resilience to continue with treatment. A woman in her 30’s in the southern region, a widowed mother of seven and a single head of household, who lost her home to her diseased husband’s family was living off charity and occasional day jobs. She elaborated on the reasons for her treatment interruptions:

I live like this anyway, I don’t have anything to eat, I don’t have anything, I’m not doing anything, I try to find a job, but I am not accepted. I end up interrupting, but not because I want to, but because I’m not able to wake up and take medication every day and continue to medicate while my stomach is complaining. In my condition, how I am… my life is fragile, today I am here… but tomorrow I may not be (Chamanculo D).

### Men’s pathways to treatment interruption

#### Patient role conflicting with provider role

The most common reason the men quoted for having interrupted an ongoing ART was difficulty of combining their provider role with being a patient. Men working in odd jobs and in trade feared losses of earnings due to waiting times at clinics.

For us men the issue is time, just one day worse for us sometimes. We are traders, losing a day for us is a big loss. (male, Nampula G)

The work-related absences for travel or work in their own distant fields lasted several months, even more than one year at a time, during which men were unable arrange continuation of treatment and due to anticipated stigma would not share with their employer their condition.

I interrupted the treatment not on my own accord, due to work, a mission of service, as you know these [projects] really are far from here. Sometimes they were in the bush for a week or a month, sometimes they moved to another district just like that. Practically I was confused, I didn’t get home for about six months. (male, Nicoadala 4)

#### Persistent denial, doubt, and shame

Denial, doubt, and shame of HIV, as manifestations of internalized stigma were common among men who interrupted their treatment. This was particularly strong among those men who did not start the prescribed treatment at all, did not disclose their diagnosis even to their family, but explained their symptoms with another diagnosis, bad spirits, or witchcraft.

A 31-year-old married man from the central region denied his HIV diagnosis from the start. He had sought care due to headache, was diagnosed with HIV but had doubted and resisted treatment for two years at the time of the interview. He had not disclosed the diagnosis to anyone, not even to his wife, who according to him had remained HIV negative. The questions about his HIV diagnosis made him angry:

Am I being questioned? What? Like *pah*, they [health staff] too insisted on me that it’s better *pah* medicate, I ah, I’m not going to medicate something that I *pah* don’t have, I’m not feeling pain *pah*. (male, Namacurra K)

Some men felt such a strong shame of their diagnosis that they would not tell anyone, not even their own family. A man in his twenties from central region who did not trust his wife to keep the secret explained:

Whoa!!! Person is person, even being together with me here, she [wife] can start to spread out there, inform friends and such and then I start to see it. Oh!!! This can be a little weird. (male, Morrumbala B)

He did, however, start treatment in hiding from his wife, and gave the facility a wrong address to avoid home visits. He further interrupted his therapy for over two months when he recognized a neighbor working in the health facility where he collected his medicine. He only resumed treatment by arranging a nurse from the clinic to bring ARVs for him privately, in secret. He explained the reason to interrupt his treatment:

So, when I saw that neighbor there, I felt so ashamed, and I started to distance myself from there [the hospital] that very day. I started to see *txa*… for me better to stop there, this neighbor of mine, if he knows, then things might not go well for me. In fact.., I don’t like this information to spread to many people. (male, Morrumbala B)

#### Isolation, depression, vulnerability

There were also a few clearly vulnerable men among the defaulters, suffering from low self-esteem, hunger, or depression. This deeply internalized stigma was linked to loss of status as breadwinner and provider, or abandonment by kin family due to HIV. A 32-year-old man had returned to live with his mother after his girlfriend had died, and he had been diagnosed with HIV. He said that the year and a half of living with his illness had been a struggle to find work as day laborer when he is weak. His hunger felt worse with the ARVs, and he interrupted his treatment for several months (Nampula H). Another man had been without treatment for two years before the interview. He was apathic, seemingly depressed, and spoke about the tragedy that caused him not to continue his medication. His wife had been the first to be diagnosed, and his own family had abandoned him after his diagnosis. He had no willingness to live in this situation of isolation from his kin family (male, Nicoadala 13).

## Pathways to adherence

**Fig 3:**
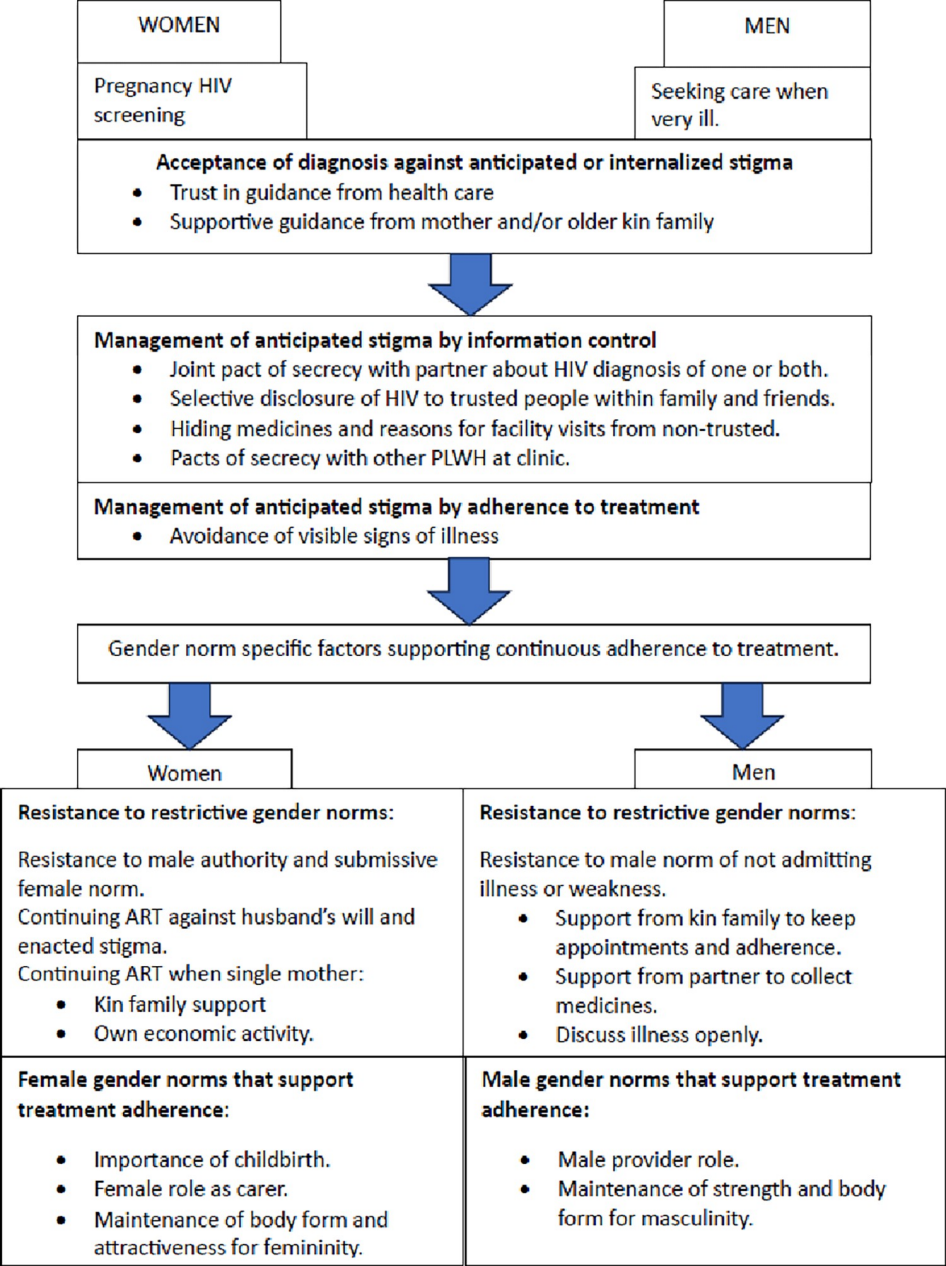
Gendered pathways to treatment adherence under HIV stigma.

The primary condition for treatment adherence for both men and women was their acceptance of the diagnosis. This meant resistance to the anticipated stigma they perceived to be prevalent in the community. A straightforward acceptance occurred most when women were diagnosed during pregnancy and when men, or non-pregnant women, sought health care with severe symptoms of illness. In these situations, both women and men generally trusted the guidance of the health care system and started treatment even if they feared stigmatization. Their gender roles supported the decision: women referred to their responsibility for the health of the unborn baby while men wanted to secure their health and strength and reverse the wasting and pain caused by the opportunistic infections. If in doubt both sought advice from their mother or other older relatives. However, after the initial start maintenance of adherence meant both managing the stigma and balancing between supportive and restrictive gender norms withing partner relations and within the context of unequal economic opportunities. The interaction between gender roles and HIV stigma in women and men’s pathways to adherence are summarized in Fig 3.

### Women’s pathways to adherence

#### Acceptance of diagnosis by partner

Most women active on treatment got their diagnosis when pregnant and accepted treatment despite initial shock, shame or fears. The motivation to have a healthy child was keeping the treatment through pregnancy, and the need to be able to care for children thereafter. If partner was also accepting the diagnosis and treatment, the adherence to treatment remained.

No, we [me and husband] didn’t suspect it [HIV] because we never got sick, that was more because of the pregnancy that I found out and he [husband] also came and took the test and found out. It was easier because we had counseling before, without that, oh it would be very difficult for me to explain it to him. (female, Chamanculo 7).

Women were generally concerned over husband’s reactions and greatly relieved if their husbands were understanding and supportive of the diagnosis and treatment. This mostly occurred when both the wife and the husband found out about their positive HIV diagnosis together, or when the husband had been hiding his treatment from the wife before she got hers. A 42-year-old woman from the southern region counted how she disclosed her diagnosis at the time of her first pregnancy to her husband, and got a surprise response from him:

I didn’t even have to convince him, I don’t even know how it went, I just know that he suddenly came with documents also saying that he was medicating in another hospital.–There was no shock, no. It became clear to me that it looked like he was the first to medicate before me. He was just afraid to tell me, but when I went to tell him, I didn’t see any remorse in him, no. Yes, I believe he didn’t feel remorse, he didn’t say anything, he just said the right thing is to medicate, let’s medicate (female, Kamavota M).

Against women’s fears many serodiscordant partners also were supportive and stayed in the relationship. A 33-year-old mother of six in the northern region told her story of growing from her internalized stigma and shame of the diagnosis and treatment interruptions to adherence with the support of an *activista* and her second husband. She had married young and had her first child at the age of 12. She was diagnosed at the age of 31 when she got very sick and was hospitalized. Initially she was in shock and denial of the HIV diagnosis and recalls feeling humiliated and ashamed. She disrupted her treatment for several months on a trip due to her shame. An *activista* helped her to restart treatment in a different facility as she feared being scolded if going back to the first facility after defaulting. She later found a new partner but was afraid and ashamed to disclose her status. She managed to hide her condition from him for a whole year until he discovered her medicines. The partner tested negative and to her surprise and relief accepted Pre-Exposure Prophylactic treatment to stay and to support her to continue her treatment (Nacala D).

#### Resisting restrictive gender norms and stigma

The common factor for women to stay adherent was to have a trusted confidant who would provide moral support in case they ran into a conflict with the partner about the HIV diagnosis. Most often women sought guidance and support from their own mothers, or older siblings in their doubts and fears and decisions about diagnosis and treatment. Economic support from kin family was particularly important if the partner had separated due to the HIV diagnosis. An example is a 21-your-old single mother from the northern region who accounted her mother’s and her ex-husband’s reactions to her disclosure of her HIV diagnosis, and how she has remained on treatment:

I told my mother’s folks, I told my husband, but he didn’t believe me, wanted to take me to another hospital to confirm. My mother didn’t agree with him and said that I should start taking medicine. Husband said that “I don’t have the disease, you do, so stay alone” and he left the next day… My mother always gave me strength, she reminded me of the day of my appointment and of taking the pills (female, Nacala 3).

A 32-year-old woman from the southern region explained how she was diagnosed at the age of 25 asymptomatic in her first pregnancy, and her husband tested negative. The husband reacted to her diagnosis by leaving her. She continued her HIV treatment despite the rejection by husband with the support of her mother. She disclosed her HIV status when meeting her second husband several years later, who to her relief stayed with her, testing regularly.

The second child already belongs to the other husband… we were dating when I spoke to him… I spoke well: ’before we do anything’… ’this disease cannot be hidden’. He said that´ I don’t have a problem, you’ll stay with me… I don’t have a problem´.. He took the test too, it was negative, always negative. And then I had the second child (laughs),… I wasn’t afraid anymore. (female, Kamavota 5)

Support from health professionals was also acknowledged by women to help them contest the expected obedient role and to maintain treatment against their husband’s will. A 28-year-old mother of two from central region who was diagnosed asymptomatic in HIV-screening in dental care explained:

My first husband forbade me to take medicine, but I did not listen to him. I was told at the health service not to listen to him, this is my health. I then separated from him. Now this new husband of mine allows me to go to the treatment without any problem. There was no interruption, even with that first husband, even though he forbade me, I always came to pick up the medication, even if he didn’t want [me] to (female, Morrumbala 10).

Ability to gain economic independence was a way for single women to work against the restrictive norm of depending on men’s income and against the stigma of the disease. A single mother of three who also cared for an orphan, had been adherent for 13 years despite being a widow of her first husband, and abandoned by the second. She sustained her children working a small plot of land and with her sister as a confidant. She did not feel shame of being on treatment:

I am not afraid of [anyone recognizing me at the clinic] because I know that it is my health that is at stake, and for the better I have to continue to medicate and not be ashamed of anyone (female, Morrumbala 9).

#### Treatment as stigma management

The few single childless young women in the study sample were particularly concerned of disclosure to anyone outside a few trusted confidants in the family, expressing strong anticipated stigma of their HIV status. A 22-year-old single childless woman in southern region was not initially ready to tell anyone, not even her own family of her diagnosis, due to shame. She waited several months before starting her treatment, not wanting to believe the result. When she started, she only disclosed it to one person in her family. The shame and anticipated stigma developed into an incentive to strict adherence. She took meticulous care of taking her medication in fear of any visible signs of disease but was also concerned of keeping the medicine in hiding. The female gender norm of keeping an attractive appearance worked in favor of the treatment in her case.

It is really that fear of appearing unwell in front of people because people just see those lesions, see your face, people can already see that you are sick. But me, I live with a lot of people, but nobody knows, and nobody sees that in me, because now I’m taking medicine (female, Kamavota E).

### Men’s pathways to adherence

#### Experience of severe illness and effectiveness of treatment

The adherent men had a common history of having received their diagnosis while seeking care when quite ill. The men talked about their suffering of devastating pains, weight loss, skin changes, continuous malaria, fevers before they sought care. The HIV diagnosis came as a surprise to most, while a few had suspected HIV.

Malaria was constant, high blood pressure was constant, I almost went crazy. (male, Nacala F)

I was sick, seriously ill, headache, stomachache, diarrhea, and high fevers and when I came here the consultation never got better, I ended up with healers, but I never got better. (male, Morrumbala 12)

My head was a little sick, it hurt a lot,.. and I immediately suspected it was HIV.. I had been with women a bit. (male, Kamavota 1)

The pivotal issue for treatment adherence was their own acceptance of the diagnosis and treatment which was strengthened by improvement of health and relief of symptoms with ART.

Experience of treatment helping to regain strength was an important incentive for men who valued their provider role. While clinic appointments could not always be easily fitted to work schedules and travel, the experience of recovering from symptoms with ART became a strong incentive to find ways to comply. Treatment provided the possibility to continue working and supporting the family.

My responsibility, as a man managing the house fund, decreased, because I was sick because I was no longer able to do activities to cover the expenses of the home. But when I started to medicate my strength recovered and now I am already starting to support at home. (male, Morrumbala 8)

I didn’t even have strength anymore when I was starting [the treatment]. At least these days I manage to go to the fields, to do the work that way. (male, Morrumbala K)

#### Resisting male norms and stigma with support from family and partner

Selective disclosure of diagnosis against male norm of not discussing illness was the most common form of resisting shame and guilt of illness. Men sought advice and support from their birth family, mother and older siblings informing them of their diagnosis. All who had disclosed to their family also accounted having received advise to start treatment as well as reminders of their clinic appointments and compliance.

My mother and my brothers are forcing me to undergo treatment and they come to my house when they see me that I went to the hospital, they always come home to check if I have already taken pills (male, Nampula 4).

Men who opened up to their wives at the time of diagnosis did not report their spouses raising arguments, even when the wives were HIV negative. The wives kept testing regularly, helped husbands to remember appointments, and even collected medicine for them.

I spoke to my wife, she said aha, no problem, we can take medicine, take it without a problem. She went to do the test, didn’t have anything. She didn’t react at all, didn’t say anything. She just said that she’s going collect that thing, the pills, so I can stay alive (male, Morrumbala I).

Discussing HIV outside of the trusted family circle was rare among men, but those who opened up to friends or neighbors all found this to support their ability to adhere. 26-year-old man from central region was diagnosed at the age of 23 when he already was married with one child. He recalled being hesitant to disclose to his wife but did so from concern for his family’s health. His wife remained seronegative, and they had another healthy child. He was not afraid to open up to a few friends about his status. The health service counselled him to open up and his friends supported adherence.

They [health staff] just told me that you can’t think too much, you can’t feel humiliated among your friends. Yes, you must be open about what you are. I wasn’t too sad either. I started to intervene with my friends, to play in the same way I had been playing, I didn’t show in any, any way of being a little sad, because the doctors encouraged me that you can’t be humiliated in society [for HIV]. My friends did not isolate me, they encouraged me to take the tablets as regulated by the hospital. (male, Nicoadala 6)

Family’s opinion about treatment was particularly important for those few men who had found out their HIV positive status without any symptoms of disease. A 30-year-old married man from northern region who had stayed on treatment for 10 years recalled the support of his family in normalizing his angst of the situation from the start when he tested without symptoms and was still single.

It was my free will to test because I had many girlfriends, many mistresses. So, I suspected myself because I had used any woman. I really wanted to know the state of my body but the result scared me. When I got back from the hospital, I informed my family, my mother, my brother and sister. They supported me, they gave me strength: “This is a disease, it’s not just for you. It is a disease that has come to many people. So, we recommend that you start medicating.” (male, Namacurra 10)

#### Joint secret as stigma management

Even accepting the diagnosis and treatment adherent men wanted to keep their HIV status a well-guarded secret within the family due to the concern over stigmatization by neighbors, workmates or other community members. Men pointed out forcefully that an HIV status in the family should not be disclosed to anyone beyond trusted family members. A man from the southern region explained:

Family is family… only the family can know… the population reacts badly… people with HIV/AIDS are afraid… to say a lot… so it’s better [for other people] not to know.

[Interviewer: What can happen if you catch someone who knows you here at the health center picking up HIV medication?] Nothing. Just a greeting. [Interviewer: You don’t talk to each other?] No, we keep the secret. (male, Kamavota 1)

Another adherent man referred to having developed a secrecy agreement between patients who would meet collecting their medicines at the facility on the same day. The aim was not to reveal each other in the community, even if drinking together, and to remind each other of the day to go to clinic speaking of the disease or appointments in disguised terms. He explained how he organized the group:

I talked to them and said that ‘look, my brothers, we are from the same group; the most important thing I’m talking about here is to shut up–keep quiet. When you get to the neighborhood, nothing has happened. Nobody saw anyone else!’ We are four people, yes. Even a brother–a really good one–on the 5thof the month he comes to my house like he’s ‘coming to have fun’ but his intention is to come and remind me [of the clinic appointment], yes: ‘Bro, ready, on the 10th, you need to come out on the day of the event.’ (male, Nampula 6)

## Discussion

HIV stigma is known not to be static, but dynamically reproduced in social interaction and in socially and culturally constructed categorizations; reproducing structural power relations and social inequalities [26, 57, 58]. One such power dimension is the local gender system [30], a pertinent one for a PLWH population in a generalized epidemic context. This study found that heterosexual men and women living with HIV in Mozambique acknowledged HIV stigma to be a persistent force in their every-day lives, that they actively sought to manage by controlling who has access to information of their HIV status. HIV stigma was felt differently by men and women at different stages of the care continuum where it emphasized the effect of the local gender roles on decision about treatment.

Three main patterns of interaction between HIV stigma domains and gender roles were found in the narratives of PLWH about their engagement with treatment. First, anticipated and internalized stigma enhanced the way in which local gender roles acted as a barrier to treatment engagement and adherence: women’s shame of HIV combined with their submissive role to their husband’s authority enhanced the fear of their husband’s negative reaction to the HIV diagnosis when a woman is depending on the husband’s economic support to survive. Equally, men’s strong internalized stigma of HIV combined with their masculine role expectations of not admitting weakness or illness resulted in diagnosis denial and became a barrier to engaging in treatment from the start.

Secondly there was an emerging pattern of resistance to restrictive gender norms which supported treatment adherence, and in some cases reduced felt anticipated stigma through improved self-esteem. Women with means to resist the subservient role, either with support from their kin family or through sustaining their children by their own economic activity, were able to continue treatment despite a partner’s disapproval. Some of them were able to build their self-esteem in the process, declaring no shame in their HIV status. Equally, men who were able to resist the restrictive male norms and accept and discuss their diagnosis, with support of family or their partner, were more likely to continue their treatment. While a few men even opened up to their friends about their condition most men were more comfortable keeping the information about the diagnosis and treatment strictly within the trusted family members.

The third emerging pattern were situations where gender roles were working in favor of treatment adherence and could even support HIV stigma management. This came to light in women appreciating that treatment increased their attractiveness and made it difficult for anyone to know from the looks who is living with HIV. Also, women accepted and adhered to ART in antenatal care and in breastfeeding periods, in accordance with their gender role as responsible carers of children. In their case the management of stigma was however not straightforward with the husband’s reaction to the HIV diagnosis possibly jeopardizing adherence. Equally, men who prioritized their provider role adhered to treatment that recovered their health and strength; however, continuation could be jeopardized by work requirements conflicting with clinic appointments.

Further, the findings point out the importance of another gender relation, that of PLWH with their older kin family members, most often the mother, with regards to management of stigma and to resistance to the restrictive gender norms of both men and women. While a few of the PLWH were reluctant to disclose to their kin family, those who did so found their family to be supportive, or even forcing them to adhere to medication. The role of an “HIV competent household” has been shown to be important for ART adherence particularly in relation to pediatric and adolescent patients and their caregivers [59, 60]. The older kin, particularly mothers of adult PLWH have appeared in HIV literature mainly as a caregivers of the dying and the orphans [61, 62]. In this study, mothers and older kin family members were referred to by the PLWH as the primary support and advisers regarding decisions about HIV disclosure and treatment. With universal treatment access the number of PLWH over 50 years old is expected to triple in the coming decades [63], and a larger proportion of the future cohorts of older trusted relatives of adult PLWH will be people with a long-term experience of ART. Despite high levels of stigma experience older PLWH in Uganda were ready to disclose their status in family and not afraid of opening up in the community [64]. Older PLWH in Mozambique have better care retention rates [65] and with their socially acknowledged advisory role in the family could be supported to guide the younger family members struggling with gender norms and stigma toward ART adherence.

The gender differences in HIV disclosure, care engagement and adherence were showing similar patterns as previous studies in the region [6, 8–14, 20, 66]. This study presents further nuance in the complex relationship between gender norms and HIV stigma as it changes in the life course of PLWH and affects decisions along the continuum of the treatment cascade. The gender roles and ideals of masculinity and femininity are shaped and reproduced in the group and community, and frame what is at stake for the stigmatizers and for those experiencing stigma [67]. Women saw HIV treatment to support their core feminine reproductive role but still as a threat to their ability to keep their partners, while for men the stigmatized diagnosis was primarily a threat to their masculine strength as breadwinners, family authorities and lovers. The intersectionality of HIV stigma and gender inequality suggests the resilience of stigma to education and awareness raising efforts targeted at individuals, without efforts to change the structural basis of inequality that stigma serves to maintain [34, 68]. An illiterate young woman married off as a teenager and infected by her husband will not be able to put the information of her rights into use without a structural change in her circumstances and opportunities. Yet, a young woman abandoned by her partner due to her HIV diagnosis can resist stigma and maintain her self- efficacy with support from an older kin family member.

Stigma of HIV continues to be a great challenge in the lives of both men and women living with HIV in Mozambique. This study illuminates mechanisms how living under restrictive gender norms and HIV stigma can, in combination with their economic circumstances, and particularly due to sudden or traumatic changes in life situations, cause PLWH to interrupt their treatment, and how they find their way back, even repeatedly [69]. ART adherence under stigma is a negotiation influenced by the prevailing gender norms and economic constraints, therefore interventions aiming to improve adherence will need to take into account the power dynamics of the social structures and their interaction with stigma. In contexts where the gender dynamic is a pertinent driver of HIV stigma, some interventions have proven to enhance treatment adherence based on sensitivity on the existing gender roles, such as women’s role in reproduction and child care [43, 44, 70]. Joint testing of partners in antenatal care can help avoid the discussion and potential conflict over who had the infection first. Further, differentiated models of care such as multi-month dispensing of ARVs can support men and single women in their provider role to have easier access to medication; despite the larger rattling bottles being more difficult to hide. However, supporting the emerging resistance to restrictive gender norms is more complex to do within the health system alone. Good examples of gender transformative interventions are found in influencing masculine norms in reducing gender-based violence [71] and enhancing women’s health and empowerment [72].

### Limitations

This paper presents a targeted analysis of data from a parent study with a wider scope into reasons of default and adherence. Both active and defaulted patients were interviewed, however selection was purposeful to examine the phenomenon, not to be representative for the population. Selection of defaulters was limited to those that the *activistas* knew of; this could introduce a bias towards those who were returning to care after interruption. Defaulters whose address or phone number were not in the clinic records could not be traced and might have given different narratives.

The semi- structured interview guide was not designed specifically for probing linkages of HIV stigma and gender roles; while questions related to both themes were part of the interviews deep diving into these was not included and the level of detail provided depended on the interviewees willingness to be open about their treatment narrative. For instance, gender-based violence was not part of the interview themes and no interviewees volunteered information about it, while from other studies it is known to be common [73]. Awareness of the social and cultural context assisted in interpreting the narratives.

Also, no other gender identities than self-identified men and women were included in the parent study, which guided the research question to be limited to heterosexual PLWH. However, the open-ended nature of the questions provided opportunities for interviewees to discuss thematic elements of both stigma and gender roles that could be inductively coded from the interview material and field notes, and example pathways to adherence and default demonstrated.

Respondents could consider the researchers to be linked to the health facility resulting in social desirability bias in their answers. They were informed by the interviewers that their willingness, or lack of it, to participate in the study would have no impact on their treatment in the health facilities. The interviewees were also faced with questions about their motivations and actions, that they might not have articulated before and therefore not willing or able to discuss at length. Yet, many interviewees, especially the defaulted ones, reflected after the interviews that they appreciated the interview very much as it was the first time they could speak about such a sensitive issue with someone who listened to them without expressing judgment, or offering advice. Hence it can be deduced that there was very little desirability bias towards acceptable answers to health workers on the interviewees’ side but rather relief to have someone to talk to without fear of judgement. Interviewers were also conscious that social desirability bias could play a role in interviewees willingness to speak on details that could be considered against the local social norms, particularly if their partner or other family member of authority was within hearing distance. Insulation from others was intended but not always possible in interviews conducted at respondents’ homes, which was then reflected in the study notes. Further, MBS who interviewed most of the defaulters is trained in using psychoanalytical techniques in interviews. She was constantly self-reflecting why questions were asked, how she felt about the answers, and whether she at any point was feeling resistance to bias arising in her, noting these in the study notes.

Part of the interviews were conducted through interpreters or with activists acting as translators from the local language which added an additional layer of filtering or interpretation of the responses, and some detail of the answers could be missed. The interviewers asked clarificatory questions to the translator when they felt that the interpretation was too short in relation to the length of explanation given by the respondent in order to fully capture the answer.

The analysis provides an overall indicative picture of issues of concern for adherence among the interviewed sample from seven districts in Mozambique, and it is not aiming to be generalizable to other contexts. The proposed linkages would require further examination through targeted qualitative work into how men and women navigate under the local gender roles and HIV stigma.

It is to be noted that the research team for the paper were all women; one Mozambican and three people with European heritage. Two of the Europeans were long term residents of Mozambique. We self-consciously reflected on our positions such as our ethnic heritage and gender when discussing the findings, results, and conclusions of the paper.

## Conclusions

The findings point to several conclusions for care and support for people living with HIV. First, improving access to ART and normalizing it in the health service is not sufficient for eliminated stigma of HIV from the lives of the people living with HIV in Mozambique. Anticipated and internalized stigma was still pervasive in the lives of heterosexual PLWH and influenced their ability to adhere to treatment. Second, stigma was reproduced in PLWH’s intimate relationships and in their interactions with their families, friends, workmates, and community, which in turn were influenced by the power gradients of gender roles and socioeconomic status. The local gender norms interacting with HIV stigma could either strengthen the PLWH ability to adhere to ART, or work against it in different phases of life, and of the treatment cascade continuum. Thirdly, the study indicates also ways how PLWH resisted restrictive gender norms and managed stigma for the benefit of treatment adherence. Consequently, the differentiated modes of delivering ART could benefit from further tailoring to the specific needs of the clients at risk of treatment interruption, to alleviate the effect of stigma by aligning with an adherence-supportive gender norm at an appropriate phase of life, or to support the PLWH’s resistance to the restrictive gender norms to remain on treatment.

A more detailed understanding of the mechanisms by which HIV stigma and gender norms interact in each context could facilitate developing improved ways of counteracting effects of stigma on HIV treatment effectiveness among heterosexual patients in generalized epidemics like in Mozambique. Sensitivity to the nuances of gender roles and what is at stake for men and women in their context and life situation should be a core part of HIV stigma reduction and treatment adherence improvement strategies. Stigma reduction interventions at the individual level can help PLWH to cope but changing the gendered power structures women and men live in requires a more holistic approach beyond the health sector alone. Interventions that support treatment adherence, need to be formulated with an understanding of local gender roles, of the prevailing domains of HIV stigma and of the powers and interests that maintain the inequality perpetuated by HIV stigma, so that they can be adequately gender sensitive, and at best, transformative.

## Supporting information

S1 Checklist (DOCX)

S1 File Interview guide. (DOCX)

S1 Dataset Sample characteristics. (XLSX)

S2 Dataset Gender excerpts. (XLSX)

S3 Dataset Stigma excerpts. (XLSX)

S4 Dataset Default case narratives. (XLSX)

S5 Dataset Active case narratives. (XLSX)
