# Supplementary material for: Gender and stigma in antiretroviral treatment adherence in Mozambique: A qualitative study
Source: PLOS Glob Public Health. 2024 Jul 15;4(7):e0003166. doi: 10.1371/journal.pgph.0003166 (PMC11249256; doi:10.1371/journal.pgph.0003166)
Supplement: S1 File — (DOCX) [file pgph.0003166.s002.docx]

## Questionnaire for the patients

1. Dados individuais

**Província**:

**Distrito:**

**Unidade Sanitária:**

**Sexo:**

**Idade:**

**Estado Civil:**

**Grávida quando iniciou tratamento?** : sim não n/d

**Abandonou tratamento**: sim não

**Se abandonou tratamento, voltou ao tratamento após as visitas de apoio?**: sim não

**Tem história de abandono de tratamento anterior?** Sim não

Bom dia, muito obrigada por concordar em participar nesta pesquisa. Antes de começar, gostaria de realçar que estamos interessados na sua experiência particular e individual que é única. Assim, não tem um certo ou errado, tudo para nós é valido. Por favor, partilhe connosco tudo que passa na sua cabeça, sem ter vergonha ou achar inapropriado.

2. Concepção de saúde e doença

1. Como o Sr. / a Sra. se sente hoje, está com saúde?
2. Agora estamos a falar de qualquer doença. Quando foi a última vez que você ou um dos seus familiares esteve doente? Como se sentiu?
3. Como sabe se você ou um familiar está doente?
   1. *Perguntar sobre possíveis sintomas.*
   2. *Explorar sobre a relação entre sintomas e doença.*
4. É possível ficar doente e não ter nenhum sinal de doença / sintoma?
5. Como sente-se uma pessoa saudável em comparação com uma pessoa doente?
6. Quando ficou doente, o que fez? (*Procurar saber se foi ao médico / US / médico tradicional ou outra pessoa)*
7. E essa pessoa o que fez? (*deu remédio, reza, tratamento tradicional*)
8. Como sentiu-se durante o tratamento e depois?
9. Quando vai á US, espera o que? Como deveria se sentir depois? E do médico tradicional? (*Indagar se espera cura, alívio de sintomas*)

3. História e percepção do HIV

1. Como sabe, a nossa pesquisa é em volta do HIV. Assim, gostaríamos de saber quando foi diagnosticado com HIV?
2. Por favor, conte mais um pouco sobre as circunstâncias:
   1. Quem lhe deu a notícia e quando?
   2. Como lhe explicou? Qual foi a informação que recebeu sobre a doença?
   3. Considerou a informação suficiente e abrangente ou ficou com dúvida?
   4. No caso de dúvida, conseguiu esclarecer com o pessoal da US, com outra pessoa ou ficou com a dúvida?
3. Pode descrever para mim, por favor, o que é o HIV/SIDA? Se for possível perceber a compreensão de vírus, o que a doença faz no corpo, possibilidades de tratamento e cura.
4. Depois de receber o diagnostico, como se sentiu? Aprofundar mais, não aceitar um “normal”.
5. Na US depois de receber o diagnóstico, lhe deram algum conselho sobre como lidar com o HIV? (Explorar se receberam apoio emocional, oferta de aconselhamento, explicações a respeito da doença, informação sobre tratamento, efeitos colaterais dos ARVs)

4. Reacção da família e comunidade

1. Depois de receber a notícia que está HIV positiva/o, como se sentiu? (Procurar saber emoções como auto-estima, sentimento de culpa, medo de morrer ou do futuro, depressão etc.)
2. Partilhou este resultado com alguém?
   1. Se partilhou com alguém: com quem foi? Como que foi? Como sentiu-se depois de ter partilhado? O que ficou diferente depois de ter partilhado o seu estado? Como esta pessoa / estas pessoas reagiram? *Explorar se ficaram assustados, solidários, se afastaram etc.*
   2. Se não partilhou com ninguém: O que esperava que aconteceria se partilhasse com alguém? Como sente-se de ficar com este resultado sozinho?
3. Caso viver com esposo/a procurar saber se ele/ela esta positivo também. Como conversam sobre a diagnose e tratamento no casal? Como se sentiu com a reação do/da parceiro/a?
4. Conhece mais pessoas vivendo com HIV/SIDA? Caso sim, pode nos explicar como estas pessoas vivem com a doença? Você aprendeu alguma coisa duma outra pessoa vivendo com HIV que lhe ajudo fazer face aos desafios do diagnóstico e tratamento? Existe um meio de ajuda/ troca de experiências mútua?
5. Como a comunidade reage quando sabe que alguém é positivo? (Explorar várias frações da comunidade como grupos religiosos, líderes, pessoas jovens ou idosas,). Como se sente sabendo destas reações? Estigma? Encorajamento? Solidariedade?

5. Papeis de gênero

1. Para você, como uma mulher ideal deveria ser? Explorar especialmente os aspectos de reprodução, saúde sexual e reprodutiva e capacidade de atrair homens.
2. E qual é a imagem/visão/percepção aqui da comunidade de uma mulher ideal?
3. Mulher ideal pode estar doente?
   1. Para mulheres: Como você se sente a respeito disso?
   2. Para homens: Como se sentiria sabendo que sua esposa/namorada está com HIV?
4. Agora a respeito de homens, para si, como um homem ideal deveria ser? Explorar especialmente os aspetos de reprodução, capacidade de alimentar a família e de atrair mulheres (muitas namoradas)
5. E qual é a imagem/visão/perceção aqui da comunidade de um homem ideal?
6. Homem ideal pode estar doente?
   1. Para homens: Como você se sente a respeito disso?
   2. Para mulheres: Como se sentiria sabendo que seu esposo/namorado está com HIV?
7. Para mulheres solteiras: na sua opinião, o facto de ser HIV positiva afecta a sua chance de encontrar um bom marido?

6. Experiência com o ARV

1. Depois do diagnóstico, quando começou com o tratamento?
2. Como foi a tomada de decisão se vai começar com o tratamento ou não? Conversou com alguém além de pessoal da US? Em que esta conversa ajudou?
3. Pode me explicar, por favor como o tratamento deveria funcionar, explorar frequência, informação sobre efeitos colaterais etc.
4. Acha que a informação recebida foi suficiente? O que é que faltava?
5. Como sentiu-se depois de iniciar o tratamento? Física- e emocionalmente (efeitos colaterais físicos e choque emocional)

7. Para pessoas LTFU

1. Como foi que decidiu de interromper o tratamento com os ARV?
   1. Falou com alguém sobre esta decisão, com quem, como se sentiu depois desta conversa? Caso não, como se sentiu tomar esta decisão sozinho/a.
   2. Caso disse que “esqueceu” explorar como se sentiu quando percebeu que se esqueceu disso, se falou com alguém sobre o esquecimento, se procurou aconselhamento com activistas ou pessoal da US.
   3. Avaliar o papel das US nesta decisão perguntando sobre:
      1. Satisfação com os serviços oferecidos,
      2. Distância e custos de chegar lá,
      3. Disponibilidade e atendimento pelo staff,
      4. Tempo de espera lá,
      5. Confidencialidade do pessoal,
      6. Explicações e serviços oferecidos e
      7. Horário de funcionamento. (ranking e exploração)

Para cada item use um ranking de 1- não satisfatório até 5 – muito satisfatório e peça explicação. Use pedrinhas caso for necessário e desenhe escala no chão.

- 1. Como sente-se quando fala com o pessoal das US? *Explorar a capacidade deles de ouvir, entender sem julgar, sentiam-se apoiados ou criticados? Tiveram a possibilidade de articular as suas dúvidas ou simplesmente tiveram que ouvir instruções etc. etc.*

1. Como sentiu-se depois de interromper o tratamento? Explorar se:
   1. Houve alterações a respeito de saúde, alivio emocional,
   2. Reação de familiares e da comunidade?
2. Depois de interromper o tratamento, um activista lhe visitou? Se foi visitado por um activista, explorar:
   1. Como sentiu-se quando chegou o activista para falar consigo?
   2. Ele/ela conseguiu lhe convencer para voltar a tomar os ARVs?
   3. Como sente-se quando fala com o activista. Explorar a capacidade deles de ouvir, entender sem julgar, *sentiam-se apoiados ou criticados? Tiveram a possibilidade de articular as suas dúvidas ou simplesmente tiveram que ouvir instruções etc.*  etc.
   4. **Se voltou a tomar ARVs:** como foi que decidiu voltar ao tratamento? Explorar se o/a activista providenciou algumas informações novas, se precisava de um encorajamento ou algo similar
   5. Como sente-se depois de ter voltado ao tratamento?
   6. Olhando para traz, o que deveria ter acontecido para não interromper o tratamento?
   7. **Se não voltou ao tratamento:** como foi que decidiu não voltar ao tratamento? Explorar:
      1. Quais os prós e contras em relação ao tratamento?
      2. O activista fez algo que lhe incomodou?
      3. Falou com alguém sobre esta decisão? Quem e como sentiu-se depois?
      4. Se não falou com alguém, como foi “discussão interna”,
      5. O que deveria acontecer para voltar ao tratamento?

8. Para pessoas que continuaram com tratamento sem interrupção

1. Já está a tomar ARVs para um tempo longo, como sente-se depois de tanto tempo tomar a medicação?
2. Já pensou uma vez em terminar ou interromper? Como foi, por favor explicar mais.
3. O que fez você continuar com a medicação? Explorar papeis dos familiares, comunidade e US.
   1. Avaliar as US a respeito de:
      1. Satisfação com os serviços oferecidos,
      2. Distância e custos de chegar lá,
      3. Disponibilidade e atendimento pelo staff,
      4. Tempo de espera lá,
      5. Confidencialidade do pessoal,
      6. Explicações e serviços oferecidos e
      7. Horário de funcionamento. (ranking e exploração)
